# Comparing movement-related cortical potential between real and simulated movement tasks from an ecological validity perspective

**DOI:** 10.3389/fnhum.2023.1313835

**Published:** 2024-01-17

**Authors:** Kakuya Ogahara, Akira Nakashima, Tomotaka Suzuki, Kenichi Sugawara, Naoshin Yoshida, Arihiro Hatta, Takefumi Moriuchi, Toshio Higashi

**Affiliations:** ^1^Department of Health Sciences, Graduate School of Biomedical Sciences, Health Sciences, Nagasaki University, Nagasaki, Japan; ^2^Department of Occupational Therapy, Kanagawa University of Human Services, Yokosuka, Japan; ^3^Department of Physical Therapy, Kanagawa University of Human Services, Yokosuka, Japan; ^4^Department of Physical Recreation, School of Physical Education, Tokai University, Hiratsuka, Japan

**Keywords:** movement-related cortical potentials, actual movement task, simulated movement task, motor learning, ecological validity, electroencephalogram

## Abstract

**Introduction:**

Concerns regarding the ecological validity of movement-related cortical potential (MRCP) experimental tasks that are related to motor learning have recently been growing. Therefore, we compared MRCP during real movement task (RMT) and simulated movement task (SMT) from an ecological validity perspective.

**Methods:**

The participants performed both RMT and SMT, and MRCP were measured using electroencephalogram (EEG). EEG was based on the 10-20 method, with electrodes placed in the motor cortex (C3 and C4) and supplementary motor cortex (FCz [between Fz and Cz] and Cz) areas. This experiment examined the MRCP using Bereitschaftspotential (BP) and negative slope (NS’) onset times, and BP, NS’, and motor potential (MP) amplitudes during the task.

**Results:**

The results revealed that the SMT exhibited later BP and NS’ onset times and smaller BP, NS’, and MP amplitudes than the RMT. Furthermore, in RMT, the onset time of MRCP was delayed, and the amplitude of MRCP was smaller in the second half of the 200 times task than in the first half, whereas in SMT, there was no change in onset time and amplitude. The SMT showed a different MRCP than the RMT, suggesting that the ecological validity of the task should be fully considered when investigating the cortical activity associated with motor skill learning using MRCP.

**Conclusion:**

Ecological validity of the study should be fully considered when investigating the cortical activity associated with motor skill learning using MRCP. Moreover, it is important to understand the differences between the two methods when applied clinically.

## 1 Introduction

Motor learning is associated with practice or experience that results in relatively permanent changes in the performer’s motor skills (Schmidt and Lee, 2005). Although motor learning is essential for human development, little is known regarding the brain mechanism. One neurophysiological method to study the underlying brain mechanisms of motor learning is the movement-related cortical potential (MRCP), which is a low-frequency negative shift in electroencephalogram (EEG) recordings that occurs approximately 1.5–2 s before the onset of voluntary movement (Kornhuber and Deecke, 1964; Hallett, 1994; Do Nascimento et al., 2006; Shibasaki and Hallett, 2006). The primary generators of MRCP originate from the bilateral supplementary motor areas, bilateral pre-supplementary motor areas, bilateral cingulate motor areas, and primary motor cortex (Mackinnon, 2003; Shakeel et al., 2015). The MRCP is believed to reflect cortical processes involved in motor planning and preparation. It is one of the most non-invasive, low-cost, and time-resolved neurophysiological techniques used to investigate changes in cortical activity during motor learning. In recent years, MRCP has been used not only in research on motor learning but also to assess the efficacy of rehabilitation interventions (Wright et al., 2012b; Fromer et al., 2016).

In general, methods to study changes in cortical activity related to motor learning can be divided into cross-sectional and longitudinal approaches; MRCP was primarily used as a cross-sectional approach to compare expert and novice participants (Kita et al., 2001; Di Russo et al., 2005; Hatta et al., 2009; Mann et al., 2011; Wright et al., 2012a; Vogt et al., 2017; Skrzeba and Vogt, 2018). Experimental tasks used in these studies appeared to be simpler to perform compared with the skills that the expert groups had been practicing for years. For instance, in clay target shooting, a simple button-pressing task was used, and in kendo, wrist extension or handgrip action was employed. While these studies show clear differences between the expert and novice groups in the adapted task, they may not account for cortical processing involved in other aspects of the skill. This difference between the actual motor learning task and simulated task set up for the MRCP measurement is thought to be caused by minimizing motion artifacts and blinking caused by body movements during the measurement. Therefore, it is crucial to analyze how the ecological validity of the motor learning task affects the MRCP.

In a recent review article, Wright et al. (2011) and Olsen et al. (2021) highlighted the issue of ecological validity in MRCP experimental tasks related to motor learning. Ecological validity refers to the degree to which the behavior analyzed for research purposes resembles actual human behavior in a real-world setting (Davids, 1988; McBurney and White, 2010). Therefore, it is crucial to analyze how the ecological validity of the motor learning task affects the MRCP. Previous reports have compared simple and complex Task in MRCP; however, these comparisons were made between different movements, such as one-ball and two-ball juggling. To our knowledge, no reports have compared real movements with simplified simulated movements in the same task from an ecological validity perspective. Therefore, we aimed to compare MRCP in real and simulated tasks from an ecological validity perspective in this study.

## 2 Materials and methods

### 2.1 Participants

The participants were 10 healthy individuals (males: 4, females: 6, mean age: 22.9 ± 4.1 years) with no history of psychiatric or neurological disorders. All participants were right-handed, as assessed using the Edinburgh Handedness Inventory. This study was conducted according to the principles of the Declaration of Helsinki and was approved by the Ethics Committee of Kanagawa University of Human Services (Approval No.5-075). The research volunteers were given oral explanations and gave consent in advance regarding the purpose, method, safety considerations, and risks of the experiment.

### 2.2 Real and simulated movement tasks

**FIGURE 1:**
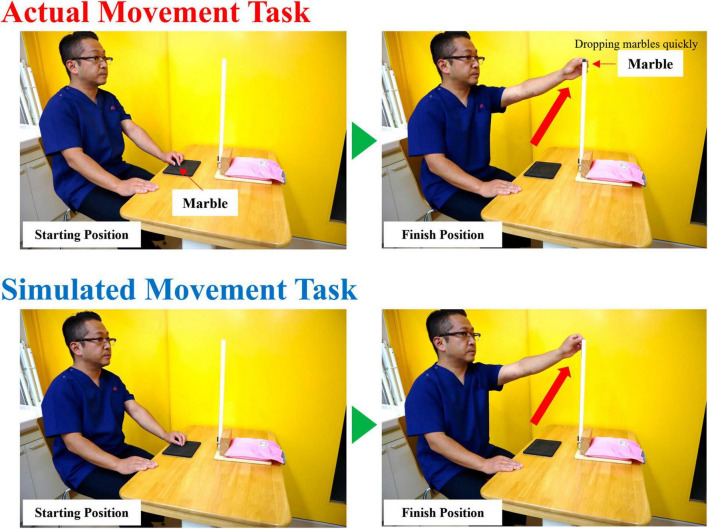
The RMT involved quickly dropping a marble (diameter: 16 mm) into a slot at eye level and height with the participant in a chair-sitting position. The SMT was a simulation of the marble dropping task. All participants performed the task with the non-dominant hand. RMT, real movement task; SMT, simulated movement task.

**FIGURE 2:**
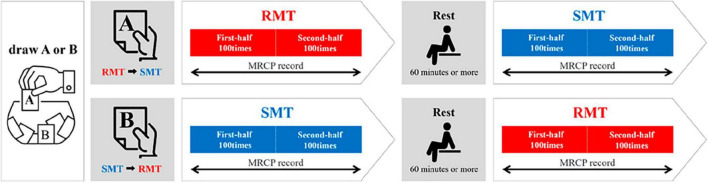
Experimental procedure. RMT, real movement task; SMT, simulated movement task.

Participants performed two tasks (real and simulated movement) (Figure 1). The real movement task (RMT) was to quickly drop a marble (diameter: 16 mm) into a slot at eye level and height, with the participant in a chair-sitting position. The simulated movement task (SMT) was a simulation of the marble-dropping task. For both the RMT and SMT, the participant was seated in a chair and started in the same position. In the SMT, a marble was not held but was quickly moved to eye level, as if it were being held. The upper limb movements and angles of the shoulder joint and other joints were similar in both tasks. During the task, all participants were instructed to use their non-dominant hand because the dominant hand is already familiar with the motion, making the detection of any significant changes during the motor learning process challenging (Yoo et al., 2013). Moreover, in routine rehabilitation, it is often necessary to train individuals to use their non-dominant hand (Sawamura et al., 2021). The experimental procedure was performed according to the flowchart depicted in Figure 2. The tasks were assigned randomly by drawing lots labeled A or B. Participants assigned to group A performed SMT after RMT, whereas those in group B performed RMT after SMT. Each task was performed in sets of approximately 25 repetitions, totaling 200 repetitions with short breaks in between. Moreover, a minimum rest period of 60 min was taken between RMT and SMT tasks.

The experiment was conducted in a quiet room in which the participants could concentrate. MRCP are easily affected by eye and body movements. Therefore, the participant was seated in an easy chair and asked to gaze at the input port to avoid blinking or eye movements. To measure the readiness potentials, the participants were instructed to avoid blinking before and after the task and observe at least a 5-s interval between tasks.

### 2.3 Electrophysiological recording

EEG was continuously recorded throughout the testing session using four 6-mm diameter, silver/silver-chloride electrodes positioned over the motor cortex, according to the International 10-20 system for electrode placement. Electrodes were placed over the left (C3) and right (C4) motor cortices overlying the hand representation and over the supplementary motor areas (FCz [between Fz and Cz] and Cz) based on previous studies (Kita et al., 2001; Hatta et al., 2009; Wright et al., 2011). An electrooculogram (EOG) was also recorded below and adjacent to the left eye to monitor both vertical (VEOG) and horizontal (HEOG) eye movements. All electrodes were referenced to the linked mastoids, and a ground electrode was placed at Fpz. The surface of the electrodes was sufficiently treated so that the resistance between the electrodes was <5 kΩ. Electrooculograms were simultaneously recorded to monitor eye movements and blinks using a recording electrode attached to the upper and lower portions of the right eyelid. The switch was placed on a desk, and the starting position for the measurement was internal rotation of the forearm. The MRCP was measured as triggered when the hand left the switch. To confirm the exact onset time, a recording electrode was attached to the anterior deltoid muscle of the left upper limb, and electromyography (EMG) was simultaneously measured. EEGs and EOGs were recorded at low (0.05 Hz) and high frequencies (50 Hz). EMGs were recorded at a low (10 Hz) and high frequencies (1,500 Hz).

### 2.4 Data analysis

After taking the measurement, the analysis software was used to automatically exclude data that had eye potentials or artifacts exceeding 50 μV. Additionally, the waveforms were checked one by one using the analysis software to eliminate any data that would affect the EEG and then re-added. More than 70 times the artifact-free waveforms were used for between-task and pre- and post-task comparisons. MRCP were analyzed for a total of 2,500 ms, from 2,000 ms at the start of the exercise to 500 ms after the exercise, using the onset time of the deltoid muscle as the reference. Bereitschaftspotential (BP) onset time, negative slope (NS’) onset time, BP amplitude, NS’ amplitude, and motor potential (MP) amplitude were used as indices. To eliminate inter-subject variability, we first averaged the EEG from 2,300 to 2,000 ms at the start of the exercise and standardized the amplitude criteria. Next, we identified the BP and NS’ onset times based on previous studies. Normally, the BP onset time is approximately 1,800–2,000 ms before the start of exercise, and the NS’ onset time is approximately 500–750 ms before the start of exercise. However, because there is a large variation due to individual differences and task difficulty, the author visually confirmed the onset time and marked the waveforms in the analysis software. The onset time and maximum amplitude during that time are calculated and displayed on the analysis software. These values were used for statistical processing. Evoked potentials and EMGs were measured using an MEB-2200 evoked potential and EMG testing device (Nihon Kohden Corp., Shinjuku, Tokyo, JAPAN), and recording and addition were processed using EPLYZER II (Kissei Comtec Corp., Matsumoto, Nagano, JAPAN).

### 2.5 Statistical analysis

A Shapiro–Wilk test indicated that the data are consistent with a normal distribution. Based on this outcome, a parametric test was used in this study. If the waveforms contained artifacts greater than 50 μV or included eye potential, the MRCP data in the study were rejected. All MRCP data ([1] BP onset time, [2] NS’ onset time, [3] BP amplitude, [4] NS’ amplitude, and [5] MP amplitude) were statistically analyzed using two-way repeated measures ANOVA with “location” (C3, Cz, C4, and FCz) and “task” (RMT and SMT) as factors to examine the effects of task and location on MRCP. In addition, we added the factor of “division” (first- and second half of the 200 times task) to the two-way ANOVA and used a three-way repeated measures ANOVA to examine its effect on MRCP between the first and second half. Simple main effect analysis with Bonferroni correction was conducted if the interaction effect is significant in both the two-way and three-way ANOVA. In all analyses, statistical significance was set at *p* < 0.05. All analyses were performed with statistical analysis software (SPSS version 22.0, IBM, Armonk, NY, USA).

## 3 Results

**FIGURE 3:**
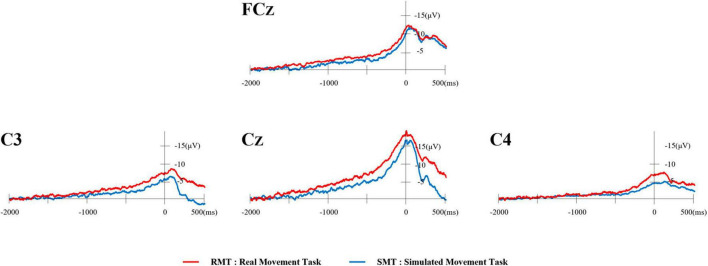
Typical waveforms of MRCP from one participant. The red waveform represents the RMT and the blue waveform represents the SMT. MRCP, movement-related cortical potential; RMT, real movement task; SMT, simulated movement task.

Typical waveforms of the MRCP for each location evaluated from one participant are shown in Figure 3. The red waveform represents the RMT, and the blue waveform represents the SMT.

**FIGURE 4:**
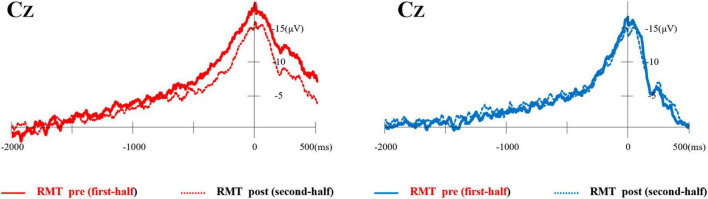
Typical waveforms of MRCP from one participant compared the first and second half of each task at Cz. The left side shows the RMT and the right side shows the SMT. MRCP, movement-related cortical potential; RMT, real movement task; SMT, simulated movement task.

Typical MRCP waveforms from one participant comparing the first and second half of each task at Cz are shown in Figure 4. The left side shows the RMT, and the right side shows the SMT. Table 1 lists the average onset time (ms) and amplitude (mV) of BP, NS’, and MP in each task.

**TABLE 1:** Mean onset times (in ms) and amplitudes (in μV) of BP, NS’, and MP in the MRCP were measured during the real and simulated movement tasks.

Electrode location	Components	Group					
		Real Movement Task			Simulated Movement Task		
		First-half	Second-half	Total (200 times)	First-half	Second-half	Total (200 times)
C3	BP onset time (ms)	1905.8 ± 233.09	1785.84 ± 186.86	1845.82 ± 214.62	1409.08 ± 286.19	1348.88 ± 281.82	1378.98 ± 278.16
	NS’ onset time (ms)	437.32 ± 39.95	426.98 ± 40.26	432.15 ± 39.40	326.56 ± 53.41	300.82 ± 48.99	313.69 ± 51.60
	BP mean amplitude (μV)	2.72 ± 0.86	1.86 ± 0.78	2.29 ± 0.91	1.40 ± 0.69	1.30 ± 0.57	1.35 ± 0.62
	NS’ mean amplitude (μV)	4.22 ± 1.36	3.00 ± 0.93	3.61 ± 1.30	2.64 ± 0.60	2.60 ± 0.57	2.62 ± 0.57
	MP peak amplitude (μV)	3.24 ± 1.53	1.66 ± 0.68	2.45 ± 1.41	1.74 ± 0.74	1.72 ± 0.70	1.73 ± 0.70
Cz	BP onset time (ms)	1921.10 ± 192.26	1773.07 ± 194.62	1851.61 ± 197.30	1421.24 ± 268.99	1365.14 ± 255.25	1393.19 ± 256.83
	NS’ onset time (ms)	428.24 ± 52.06	415.58 ± 51.37	421.91 ± 50.75	323.08 ± 50.02	305.43 ± 47.00	314.25 ± 48.00
	BP mean amplitude (μV)	4.62 ± 1.26	3.86 ± 0.75	4.24 ± 1.08	2.64 ± 0.98	2.51 ± 0.97	2.57 ± 0.95
	NS’ mean amplitude (μV)	9.16 ± 2.82	6.71 ± 1.71	7.93 ± 2.59	2.60 ± 0.98	2.51 ± 0.87	2.55 ± 0.87
	MP peak amplitude (μV)	7.93 ± 3.12	3.70 ± 0.76	5.81 ± 3.09	2.70 ± 0.85	2.51 ± 0.82	2.6 ± 0.81
C4	BP onset time (ms)	1943.06 ± 168.95	1771.26 ± 168.75	1857.16 ± 186.41	1404.82 ± 243.57	1374.44 ± 245.45	1389.63 ± 238.50
	NS’ onset time (ms)	453.54 ± 36.17	436.4 ± 30.61	444.97 ± 33.78	352.04 ± 46.97	341.70 ± 44.50	346.87 ± 44.84
	BP mean amplitude (μV)	3.40 ± 1.31	2.72 ± 0.82	3.06 ± 1.12	1.30 ± 0.58	1.24 ± 0.58	1.27 ± 0.57
	NS’ mean amplitude (μV)	5.14 ± 2.38	2.74 ± 0.94	3.94 ± 2.15	3.11 ± 0.72	3.09 ± 0.69	3.10 ± 0.70
	MP peak amplitude (μV)	3.08 ± 1.3	0.65 ± 0.38	1.86 ± 1.55	1.37 ± 0.89	1.30 ± 0.9	1.33 ± 0.87
FCz	BP onset time (ms)	1907.54 ± 202.42	1686.98 ± 195.05	1797.26 ± 224.12	1396.4 ± 307.25	1365.48 ± 273.96	1380.94 ± 283.75
	NS’ onset time (ms)	455.2 ± 47.36	426.52 ± 37.42	440.86 ± 44.07	371.64 ± 50.48	369 ± 49.63	370.64 ± 48.73
	BP mean amplitude (μV)	4.60 ± 1.39	3.80 ± 0.90	4.20 ± 1.21	2.14 ± 0.70	2.14 ± 0.73	2.14 ± 0.69
	NS’ mean amplitude (μV)	6.80 ± 2.39	4.98 ± 1.38	5.89 ± 2.11	5.89 ± 1.33	6.00 ± 1.41	5.94 ± 1.34
	MP peak amplitude (μV)	4.92 ± 2.32	3.58 ± 1.31	4.25 ± 1.96	3.80 ± 1.18	3.80 ± 1.21	3.80 ± 1.16

BP, Bereitschaftspotential; MP, motor potential; MRCP, movement-related cortical potential; NS’, negative slope.

### 3.1 The effect of difference on BP onset time and NS’ onset time between tasks

**FIGURE 5:**
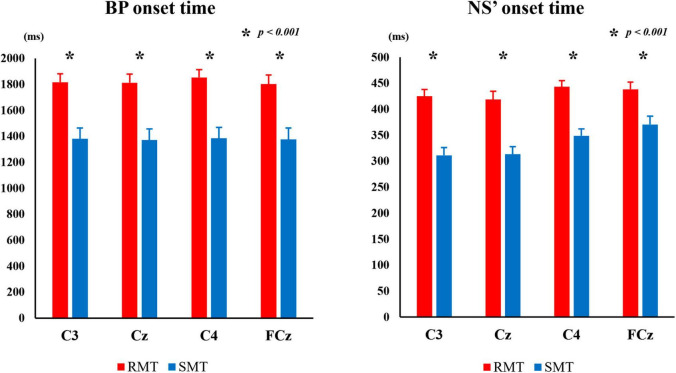
The average BP onset time and NS’ onset time during the RMT and SMT at C3, Cz, C4 and FCz. MRCP, movement-related cortical potential; BP, Bereitschaftspotential; NS’, negative slope; RMT, real movement task; SMT, simulated movement task.

Figure 5 shows the average of BP and NS’ onset times during the RMT and SMT at each location.

The two-way repeated measures ANOVA revealed significant interaction between the effects of “location” and “task” on BP and NS’ onset times (F_(3_, _27)_ = 4.834, *p* < 0.01, *partial*η^2^ = 0.349 and F_(3_, _27)_ = 4.414, *p* < 0.05, *partial*η ^2^ = 0.329, respectively). Simple main effects analysis showed that the RMT exhibited significantly longer BP onset time than the SMT in C3 (F_(1_, _9)_ = 163.987, *p* < 0.001, *partial*η^2^ = 0.948), Cz (F_(1_, _9)_ = 176.086, *p* < 0.001, *partial*η^2^ = 0.951), C4 (F_(1_, _9)_ = 161.493, *p* < 0.001, *partial*η^2^ = 0.947), and FCz (F_(1_, _9)_ = 159.370, *p* < 0.001, *partial*η^2^ = 0.947), and the RMT also exhibited significantly longer NS’ onset time than the SMT in C3 (F_(1_, _9)_ = 131.316, *p* < 0.001, *partial*η^2^ = 0.936), Cz (F_(1_, _9)_ = 55.126, *p* < 0.001, *partial*η^2^ = 0.860), C4 (F_(1_, _9)_ = 76.983, *p* < 0.001, *partial*η^2^ = 0.895) and FCz (F_(1_, _9)_ = 39.671, *p* < 0.001, *partial*η^2^ = 0.815).

### 3.2 The effect of difference on BP amplitude and NS’ amplitude and MP amplitude between tasks

**FIGURE 6:**
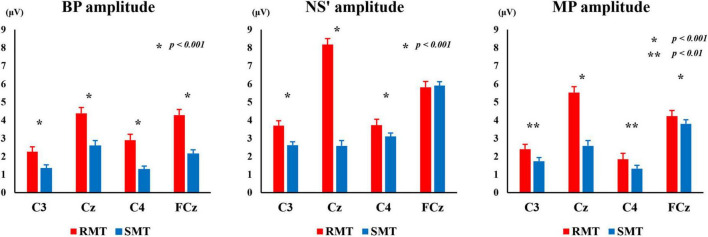
The average of BP, NS’ and MP amplitude during the RMT and SMT at C3, Cz, C4 and FCz. BP, Bereitschaftspotential; NS’, negative slope; RMT, real movement task; SMT, simulated movement task.

Figure 6 shows the average BP, NS’, and MP amplitudes during the RMT and SMT tasks at each location.

The two-way repeated measures ANOVA revealed statistically significant interaction between the effects of “location” and “task” on BP, NS’, and MP amplitudes [F_(3_, _27)_ = 18.333, *p* < 0.001, *partial*η^2^ = 0.671 (BP amplitude), F_(3_, _27)_ = 150.440, *p* < 0.001, *partial*η^2^ = 0.944 (NS’ amplitude), F_(3_, _27)_ = 93.709, *p* < 0.01, *partial*η^2^ = 0.912 (MP amplitude)]. Simple main effects analysis showed that the RMT exhibited significantly larger BP amplitude than the SMT in in C3 (F_(1_, _9)_ = 47.139, *p* < 0.001, *partial*η^2^ = 0.840), Cz (F_(1_, _9)_ = 729.000, *p* < 0.001, *partial*η^2^ = 0.988), C4 (F_(1_, _9)_ = 63.562, *p* < 0.001, *partial*η^2^ = 0.876), and FCz (F_(1_, _9)_ = 270.449, *p* < 0.001, *partial*η^2^ = 0.968). Furthermore, the RMT showed significantly larger NS’ amplitude than the SMT in in C3 (F_(1_, _9)_ = 72.099, *p* < 0.001, *partial*η^2^ = 0.889), Cz (F_(1_, _9)_ = 193.846, *p* < 0.001, *partial*η^2^ = 0.956), and C4 (F_(1_, _9)_ = 32.152, *p* < 0.001, *partial*η^2^ = 0.781). The RMT also exhibited significantly larger MP amplitude than the SMT in C3 (F_(1_, _9)_ = 18.812, *p* < 0.01, *partial*η^2^ = 0.676), Cz (F_(1_, _9)_ = 144.373, *p* < 0.001, *partial*η^2^ = 0.941), C4 (F_(1_, _9)_ = 16.714, *p* < 0.01, *partial*η^2^ = 0.650), and FCz (F_(1_, _9)_ = 36.413, *p* < 0.001, *partial*η^2^ = 0.802).

### 3.3 The effect of difference on BP onset time and NS’ onset time during RMT and SMT between the first and second half of the 200 times task

**FIGURE 7:**
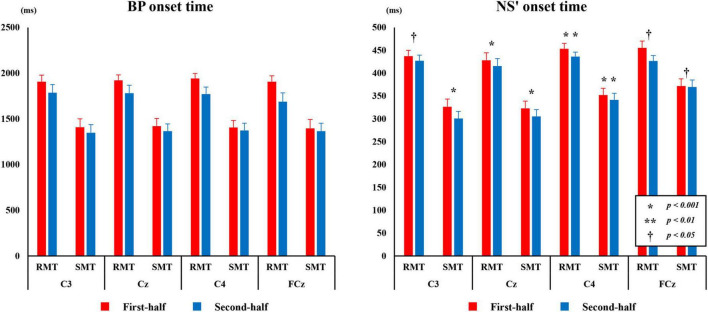
The average BP, and NS’ onset time from C3, Cz, C4, and FCz compared the first and second half of each task. BP, Bereitschaftspotential; NS’, negative slope.

Figure 7 shows the average BP and NS’ onset times during the RMT and SMT between the first and second half at each site.

#### 3.3.1 BP onset time

The three-way repeated measures ANOVA revealed a statistically significant interaction between the effects of “location” and “division” on BP time (F_(3_,_27)_ = 15.753, *p* < 0.001, *partial*η^2^ = 0.636). Simple main effects analysis showed that the BP onset time for both RMT and SMT of the first-half division was significantly longer than that of the second-half division in C3 (F_(1_, _9)_ = 18.672, *p* < 0.01, *partial*η^2^ = 0.675), Cz (F_(1_, _9)_ = 56.979, *p* < 0.01, *partial*η^2^ = 0.864), C4 (F_(1_, _9)_ = 51.682, *p* < 0.01, *partial*η^2^ = 0.852), and FCz (F_(1_, _9)_ = 6.601, *p* < 0.05, *partial*η^2^ = 0.423).

#### 3.3.2 NS’ onset time

The three-way repeated measures ANOVA revealed a statistically significant interaction between the effects of “location,” “task,” and “division” on NS’ onset time (F_(3_,_27)_ = 4.673, *p* < 0.01, *partial*η^2^ = 0.342). Simple main effects analysis showed that the NS’ onset time for both RMT and SMT of the first-half division was significantly longer than that of the second-half division in C3(RMT: F_(1_, _9)_ = 5.394, *p* < 0.05, *partial*η^2^ = 0.375, SMT: F_(1_, _9)_ = 23.317, *p* < 0.01, *partial*η^2^ = 0.722), Cz (RMT: F_(1_, _9)_ = 17.416, p < 0.01, *partial*η^2^ = 0.659, SMT: F_(1_, _9)_ = 10.390, *p* < 0.05, *partial*η^2^ = 0.536), C4 (RMT: F_(1_, _9)_ = 66.002, *p* < 0.001, *partial*η^2^ = 0.880, SMT: F_(1_, _9)_ = 36.901, *p* < 0.001, *partial*η^2^ = 0.804), and FCz (RMT: F_(1_, _9)_ = 13.663, *p* < 0.01, *partial*η^2^ = 0.603, SMT: F_(1_, _9)_ = 7.075, *p* < 0.05, *partial*η^2^ = 0.440).

### 3.4 The effect of difference on BP amplitude, NS’ Amplitude, and MP Amplitude between the first and second half

**FIGURE 8:**
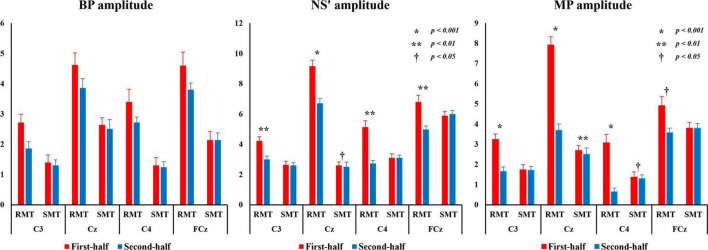
BP, NS’, and MP amplitudes from C3, Cz, C4, and FCz compared the first and second half of each task. BP, Bereitschaftspotential; MP, motor potential; NS’, negative slope.

Figure 8 shows the average BP, NS’, and MP amplitudes during the RMT and SMT between the first and second halves at each location.

#### 3.4.1 BP amplitude

The three-way repeated measures ANOVA revealed that there was a statistically significant interaction between the effects of “location” and “task” on BP amplitude (F_(3_,_27)_ = 14.406, *p* < 0.001, *partial*η^2^ = 0.615) and “location” and “division” (F_(3_,_27)_ = 14.860, *p* < 0.001, *partial*η^2^ = 0.623). Simple main effects analysis showed that the BP amplitude for RMT was significantly larger than that for SMT in C3 (F_(1_, _9)_ = 101.701, *p* < 0.001, *partial*η^2^ = 0.919), Cz (F_(1_, _9)_ = 32.758, *p* < 0.001, *partial*η^2^ = 0.784), C4 (F_(1_, _9)_ = 43.132, *p* < 0.001, *partial*η^2^ = 0.827), and FCz (F_(1_, _9)_ = 168.798, *p* < 0.001, *partial*η^2^ = 0.949). Moreover, the first-half division of BP amplitude of was significantly larger than the second-half division in C3 (RMT: F_(1_, _9)_ = 40.197, *p* < 0.001, *partial*η^2^ = 0.817), Cz (RMT: F_(1_, _9)_ = 14.780, *p* < 0.01, *partial*η^2^ = 0.622) and C4 (RMT: F_(1_, _9)_ = 26.304, *p* < 0.01, *partial*η^2^ = 0.745).

#### 3.4.2 NS’ amplitude

The three-way repeated measures ANOVA revealed that there was a statistically significant interaction between the effects of “location,” “task,” and “division” on NS’ amplitude (F_(3_,_27)_ = 11.532, *p* < 0.001, *partial*η^2^ = 0.562). Simple main effects analysis showed that the NS’ amplitude for RMT of the first-half division was significantly larger than that of the second-half division in C3(F_(1_, _9)_ = 21.620, *p* < 0.01, *partial*η^2^ = 0.706), Cz (F_(1_, _9)_ = 31.881, *p* < 0.001, *partial*η^2^ = 0.780), C4 (F_(1_, _9)_ = 22.657, *p* < 0.01, partial η2 = 0.716) and FCz (F_(1_, _9)_ = 22.627, *p* < 0.01, *partial*η^2^ = 0.715). However, the NS’ amplitude for SMT of the first-half division was significantly larger than that of the second-half division only in Cz (F_(1_, _9)_ = 8.191, *p* < 0.05, *partial*η^2^ = 0.476).

#### 3.4.3 MP amplitude

The three-way repeated measures ANOVA revealed that there was a statistically significant interaction between the effects of “location,” “task,” and “division” on MP amplitude (F_(3_,_27)_ = 18.559, *p* < 0.01, *partial*η^2^ = 0.673). Simple main effects analysis showed that the MP amplitude for RMT of the first-half division was significantly larger than that of the second-half division in C3(F_(1_, _9)_ = 30.378, *p* < 0.01, *partial*η^2^ = 0.771), Cz (F_(1_,_9)_ = 27.518, *p* < 0.001, *partial*η^2^ = 0.754), C4 (F_(1_,_9)_ = 56.410, *p* < 0.01, *partial*η^2^ = 0.862), and FCz (F_(1_,_9)_ = 9.998, *p* < 0.05, *partial*η^2^ = 0.526). However, the MP amplitude for SMT of the first-half division was significantly larger than that of the second-half division only in Cz (F_(1_,_9)_ = 21.805, *p* < 0.01, *partial*η^2^ = 0.708) and C4 (F_(1_,_9)_ = 5.444, *p* < 0.05, *partial*η^2^ = 0.377).

## 4 Discussion

### 4.1 Comparison of MRCP in actual and simulated movement tasks

In this study, we compared MRCP between a task-oriented RMT and SMT to investigate task-specific changes in MRCP from an ecological perspective. The results revealed that the MRCP began later and the amplitude of the MRCP was smaller in the SMT than in the RMT. These results are in agreement with the findings of previous reports that MRCP appear earlier in complex movements and that the amplitude of MRCP is significantly larger in complex movements (Frömer et al., 2012; Berchicci et al., 2017; Tomyta and Seki, 2020). These results suggest that RMT requires more cortical activity than SMT in planning and preparing to execute movements. In this study, the range of motion of the shoulder and elbow joints is almost the same in both the RMT and SMT. However, it should be noted that the RMT involves not only picking up the marble but also requires precise finger movements and good eye-hand coordination to place the marble accurately into the slot. In contrast, the SMT without marble holding required less cortical activity than the RMT.

In addition, when comparing the first half and second half of all experimental sessions, RMT had a longer MRCP “onset time” and decrease in amplitude in the second half compared with those in the first half. A similar trend was observed in SMT for both onset time and amplitude parameters. However, the amount of change between the first and second half division in SMT was smaller than that in RMT, and in some cases, there was no significant difference was observed between the first- and second half division in certain brain regions. In a previous comparison of MRCP between experts and novices to examine changes in MRCP with motor learning, it was reported that the onset time of MRCP was later (Kita et al., 2001; Di Russo et al., 2005; Hatta et al., 2009; Vogt et al., 2017), and the amplitude of the MRCP was smaller in experts than in novices (Kita et al., 2001; Di Russo et al., 2005; Shibasaki and Hallett, 2006; Hatta et al., 2009; Wright et al., 2012a). These results indicate that long-term training reduces the time required for planning and preparing to perform the behavior in experts. This interpretation is supported by functional magnetic resonance imaging studies showing that long-term practice leads to use-dependent changes in cortical activation such that fewer neurons are activated to perform the same task (Jäncke et al., 2000; Haslinger et al., 2004). Furthermore, a decrease in the MRCP amplitude was observed after repeated motor task training in the same participants, which may reflect a decrease in the cortical effort required to perform the task. Therefore, from this perspective, it can be inferred that in the RMT in the present study, fewer neurons were activated to perform the same task in the last 100 trials than in the first 100 trials owing to motor learning. Notably, no clear difference was observed between the first and second halves of the MRCP because the SMT was a simple task requiring little planning or preparation.

In this study, EEG measurement electrodes were placed at C3, C4, FCz, and Cz. As a result, the source of MRCP was identified as the ipsilateral primary motor cortex at C3, contralateral primary motor cortex at C4, and supplementary motor cortex at FCz and Cz. A similar trend was observed in all locations including C3, originating from the ipsilateral primary motor cortex during both tasks in this study. Shibasaki et al. (1993) performed a positron emission tomography study and found that cerebral blood flow in the cerebellum and motor cortex on the same side as the movement increased with task difficulty. Therefore, we concluded that the difficulty of the task had an impact on the results obtained from C3, which exhibited a similar trend.

### 4.2 Clinical implications

In recent years, considerable evidence has been reported in the field of rehabilitation for task-specific training, which involves repetitive, goal-directed movements that are closer to movements in the real-world setting than simple repetitive joint movements (Hubbard et al., 2009; Tse et al., 2018). It has been suggested that tasks in task-specific training should be “real-world” or situation-specific. In this study, the onset of MRCP occurred earlier in RMT compared with in SMT. Additionally, the amplitude of MRCP was larger in RMT than in SMT. These findings suggest that the RMT requires a higher level of cortical activity for planning and executing the motor movements.

Similar to the present study, a kinematic analysis of the performance of reaching for and picking up a coin from a table and reaching the exact location without a coin also reported that the task using a real object elicited a kinematically superior performance (Wu et al., 2000). The report revealed that the real-object task produced a kinematically superior performance. Overall, these findings suggest that task-specific training in clinical rehabilitation may be more effective when the tasks are real movement tasks in real-world contexts rather than simplified simulated movements.

### 4.3 Study limitations

It is important to consider several limitations of this study. First, the sample size used was relatively small. Second, pre- and post-task performance was not measured, which makes it difficult to convincingly explain the changes in MRCP between the first and second half of the 200 times task in relation to motor learning. Additionally, the present comparisons were made only on the same experimental day. The long-term effects of task repetition have not been determined yet. Therefore, it is recommended to conduct future larger-scale studies using tasks that allow the measurement of changes in performance and include long-term effects.

## 5 Conclusion

Our results suggest that MRCP in the SMT is different from that in the RMT and that the ecological validity of the task should be fully considered when investigating cortical activity associated with motor skill learning using MRCP. When applying these methods clinically, it is essential to understand the differences between the two tasks.

## Data availability statement

The original contributions presented in this study are included in this article/supplementary material, further inquiries can be directed to the corresponding author.

## Ethics statement

The studies involving humans were approved by the Ethics Committee of Kanagawa University of Human Services (Approval No. 5-075). The studies were conducted in accordance with the local legislation and institutional requirements. The participants provided their written informed consent to participate in this study. Written informed consent was obtained from the individual(s) for the publication of any identifiable images in this article.

## Author contributions

KO: Data curation, Funding acquisition, Investigation, Writing – original draft. AN: Data curation, Writing – review and editing. TS: Investigation, Writing – review and editing. KS: Supervision, Validation, Writing – review and editing. NY: Data curation, Investigation, Writing – review and editing. AH: Supervision, Validation, Writing – review and editing. TM: Data curation, Validation, Writing – review and editing. TH: Conceptualization, Supervision, Writing – original draft.
